# Simulated Microgravity Increases the Permeability of HUVEC Monolayer through Up-Regulation of Rap1GAP and Decreased Rap2 Activation

**DOI:** 10.3390/ijms23020630

**Published:** 2022-01-06

**Authors:** Shuliang Shi, Jing Li, Erzhuo Li, Wenqi Guo, Yao He, Jinpeng Wang, Yao Zhang, Lei Yue, Lijun Wei

**Affiliations:** 1 School of Life Science and Technology, Harbin Institute of Technology, Harbin 150001, China; liangss@hit.edu.cn (S.S.); yangqianusst@163.com (J.L.); s1005674406@163.com (E.L.); guowenqi0304@163.com (W.G.); hy17861120139@163.com (Y.H.); 20B928024@stu.hit.edu.cn (J.W.); zhangyao83326@163.com (Y.Z.); yuelei@hit.edu.cn (L.Y.); 2 State Key Laboratory of Space Medicine Fundamentals and Application, Chinese Astronaut Research and Training Center, Beijing 100094, China

**Keywords:** simulated microgravity, adhesions junction, HUVEC cell, Rap1GAP, VE-cadherin

## Abstract

Space microgravity condition has great physiological influence on astronauts’ health. The interaction of endothelial cells, which control vascular permeability and immune responses, is sensitive to mechanical stress. However, whether microgravity has significant effects on the physiological function of the endothelium has not been investigated. In order to address such a question, a clinostat-based culture model with a HUVEC monolayer being inside the culture vessel under the simulated microgravity (SMG) was established. The transmittance of FITC-tagged dextran was used to estimate the change of integrity of the adherens junction of the HUVEC monolayer. Firstly, we found that the permeability of the HUVEC monolayer was largely increased after SMG treatment. To elucidate the mechanism of the increased permeability of the HUVEC monolayer under SMG, the levels of total expression and activated protein levels of Rap1 and Rap2 in HUVEC cells, which regulate the adherens junction of endothelial cells, were detected by WB and GST pull-down after SMG. As the activation of both Rap1 and Rap2 was significantly decreased under SMG, the expression of Rap1GEF1 (C3G) and Rap1GAP in HUVECs, which regulate the activation of them, was further determined. The results indicate that both C3G and Rap1GAP showed a time-dependent increase with the expression of Rap1GAP being dominant at 48 h after SMG. The down-regulation of the expression of junctional proteins, VE-cadherin and β-catenin, in HUVEC cells was also confirmed by WB and immunofluorescence after SMG. To clarify whether up-regulation of Rap1GAP is necessary for the increased permeability of the HUVEC monolayer after SMG, the expression of Rap1GAP was knocked down by Rap1GAP-shRNA, and the change of permeability of the HUVEC monolayer was detected. The results indicate that knock-down of Rap1GAP reduced SMG-induced leaking of the HUVEC monolayer in a time-dependent manner. In total, our results indicate that the Rap1GAP-Rap signal axis was necessary for the increased permeability of the HUVEC monolayer along with the down-regulation of junctional molecules including VE-cadherin and β-catenin.

## 1. Introduction

The endothelium formed by an endothelial cell monolayer functions as a selective barrier between the bloodstream and tissues, preventing the leaking of small molecules, macromolecules, and cells from blood to the underlying tissue [1,2]. The significance of vascular integrity controlled by endothelial cell junctions is stressed by the fact that vascular hyperpermeability is related to diseases such as hemorrhage, chronic atherosclerosis, inflammation, and diabetes [3]. Adherens junctions are highly dynamic, and the exchange of molecules or extravasation and intravasation of white blood cells across the endothelial barrier could be achieved by regulation of the permeability of the endothelium [4]. The opening or closing of the endothelial barrier, which is dependent on the dynamics of adherens junctions, are regulated by the perivascular microenvironment, such as angiogenic cues, shear stress, or inflammatory factors, etc.

Vascular endothelial cadherin (VE-cadherin), the transmembrane glycoprotein restricted in endothelial cells, plays a key role in the regulation of adherens junctions [5]. The trans-interaction of VE-cadherin between endothelial cells could recruit β-catenin and p120-catenin to the cytosolic domain of VE-cadherin to stabilize the endothelial adherens junctions. Furthermore, the interaction of α-catenin, vinculin, and EPLIN with the cytosolic domain of VE-cadherin could mediate the connection of adherens junctions to the actin cytoskeleton to form zonula adherens and maintain the functional integrity of the vascular barrier of the endothelium by facilitating the formation of adherens junctions between endothelial cells [6,7,8,9,10,11]. The regulation of the permeability of endothelial adherens junctions involves a complex process that contains vesicular trafficking, junctional rearrangements, and accurate cytoskeletal dynamics [9]. The expression and localization of VE-cadherin stand the focal point of the permeability regulation, as it dictates the level of expression and the localization of other junctional molecules, including claudin-5, N-cadherin, and so on [10].

Availability of VE-cadherin at the plasma membrane is necessary for endothelial barrier function, as VE-cadherin internalization or down-regulation is associated with the loss of cell–cell junctions, leading to enhanced cell migration and permeability [12]. Small G proteins including Rho family members (RhoA, Cdc42, and Rac1) and Rap family molecules (Rap1, Rap2) play key roles in the regulation of the barrier function of VE-cadherin-mediated adherens junctions by working downstream of the perivascular microenvironment and mechanical stress. Hormones and agonists regulate the permeability of the endothelium through intracellular signaling events that modulate the strength of the HUVEC monolayer formed by VE-cadherin [13,14]. Meanwhile, several signaling pathways have been shown to regulate the endocytosis of VE-cadherin in clathrin-coated pits including PI3K, Src, and PAK which work together to modulate the adherens junctions formed between endothelial cells [15].

Rap subfamily small G proteins consisting of closely related members, Rap1 and Rap2, belong to the Ras superfamily. Rap proteins function as a molecular switch that shifts between GDP-bound inactivated state and GTP-bound activated state, depending on the availability of two groups of factors: Rap guanine nucleotide factors (RapGEFs) and Rap GTPase-activating proteins (RapGAP), such as other G proteins [16].

Multiple guanine nucleotide exchange factors for small GTPase Rap1 and Rap2 mediate the signaling that regulates the tightening of the endothelium [17]. Among them, C3G, also named Rap1GEF1, exchange proteins activated by cAMP (Epac) and PDZ-GEF, have been proposed to regulate the formation of adherens junctions between endothelial cells through the activation of Rap1 [17,18]. In addition to its role in the activation of PKA, cAMP is a direct activator of Epac1 and Epac2 which activate both Rap1 and Rap2 [19]. C3G and PDZ-GEF could activate both Rap1 and Rap2, whereas RasGEF1 shows specificity toward Rap2 [20,21]. Epac1 decreases the cell permeability through its guanine nucleotide exchange factor (GEF) activity toward Rap1 and also through direct effects on microtubules [22]. C3G, the first found guanine nucleotide exchange factor for Rap1, is necessary for the recovery of the EC barrier through activating Rap1, following the thrombin-induced increases of endothelial permeability [23,24].

Rap1 (Krev-1), originally identified as a gene with revertant-inducing activity on K-Ras oncogene, exists as two isoforms, Rap1A and Rap1B, regulating a variety of cellular functions including cell transformation, cell spreading, and formation of adherens junction between cells [25,26,27,28,29,30]. Rap1 was also isolated in a search for homologous genes to Drosophila Dras 3 along with Rap2 [31]. Rap1GAP, firstly isolated as a specific GTPase-activating protein toward Rap1 (Krev-1), negatively regulates the activation of Rap1 and Rap2 by promoting the intrinsic GTPase activity that catalyzes the degradation of GTP bound to Rap proteins to GDP [21,32]. The overexpression of Rap1GAPs leads to an increase in endothelial permeability by abolishing Rap1 activity [29,30]. Deletion of Rap1GAP, in fact, prevents the formation of adherens junctions between carcinoma cells [33]. Although Rap2 shares a 65% homology in amino acid sequence with Rap1, the function in the regulation of the endothelial permeability of Rap2 seems not to be consistent with Rap1 [34]. There are three isoforms of Rap2, Rap2A, Rap2B, and Rap2C, showing the major differences at the C-terminal part of the proteins, which determines different subcellular localization of them [35]. Rap2-specific effectors, including MAP4K4, MINK, TINK, and PARG, may connect Rap2 signaling to the regulation of the microfilament cytoskeleton [36].

The endothelial cells are very sensitive to microgravity and would show morphological and functional changes under a microgravity condition [37,38]. To explore whether the physiological function of the endothelium could be altered during microgravity conditions, we established a clinostat-based HUVEC monolayer permeability assay model under SMG, and the permeability of the HUVEC monolayer was detected under SMG. The physiological impact on the HUVEC monolayer of small G protein Rap1 and Rap2, as well as their upstream regulators including Rap1GAP and C3G, were investigated.

## 2. Results

### 2.1. Modulation of Endothelium Permeability by Simulated Microgravity

**Figure 1:**
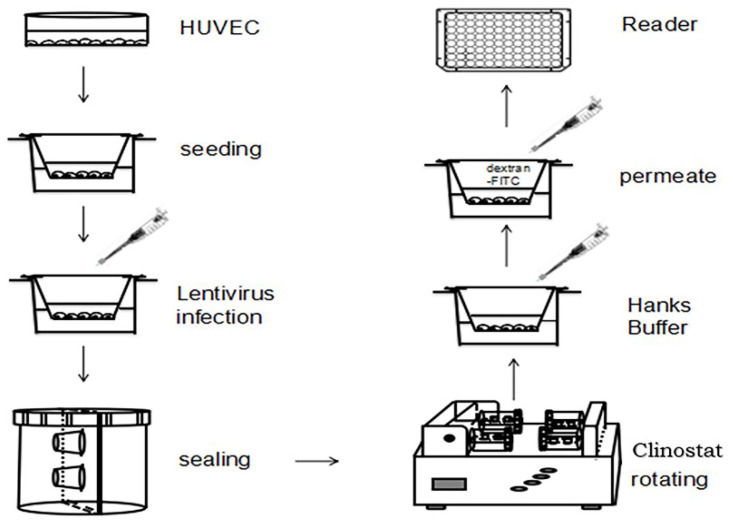
Establishment of monolayer cell permeability model under SMG.

The phenotype of HUVECs, human umbilical vein endothelial cells, has shown to be highly affected by microgravity leading to various physiological changes including an abnormal cytoskeleton and downregulated mechanic sensing proteins [38]. However, whether microgravity has effects on the physiological function of the endothelium has not been reported up until now. To investigate the potential effects of microgravity on the permeability of the endothelium, we established a monolayer HUVEC cell model under SMG by inserting a transwell chamber with pores of 3 µm in diameter and determined the effects of SMG on the permeability of monolayer HUVEC cells (Figure 1).

**Figure 2:**
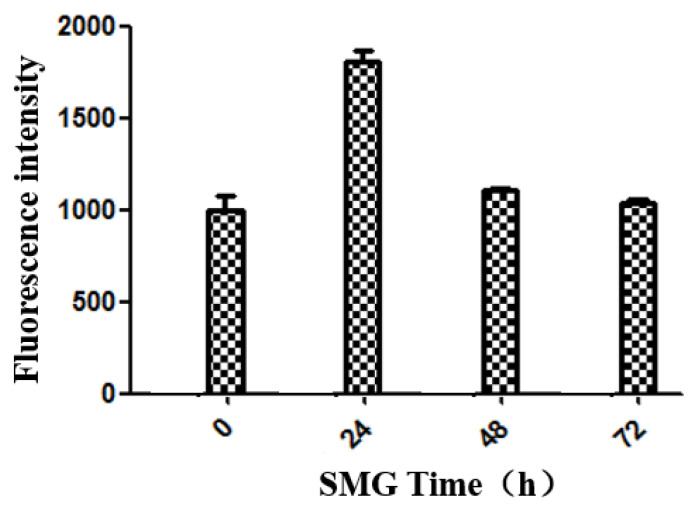
Permeability of HUVEC monolayer under SMG. HUVEC cells were plated on culture chamber and grown to confluence to form monolayer and set to 2D clinorotation. Then the permeability of HUVEC monolayer under SMG for 24, 48, and 72 h was assayed.

The results show that the permeability of the HUVEC monolayer was increased at 24 h SMG treatment and recovered to the normal level gradually until 48h after SMG (Figure 2). The increased permeability of the HUVEC monolayer suggested that the adherens junction of HUVEC cells might be decreased by SMG treatment.

### 2.2. The Activation of Rap2 Was Down-Regulated under SMG

**Figure 3:**
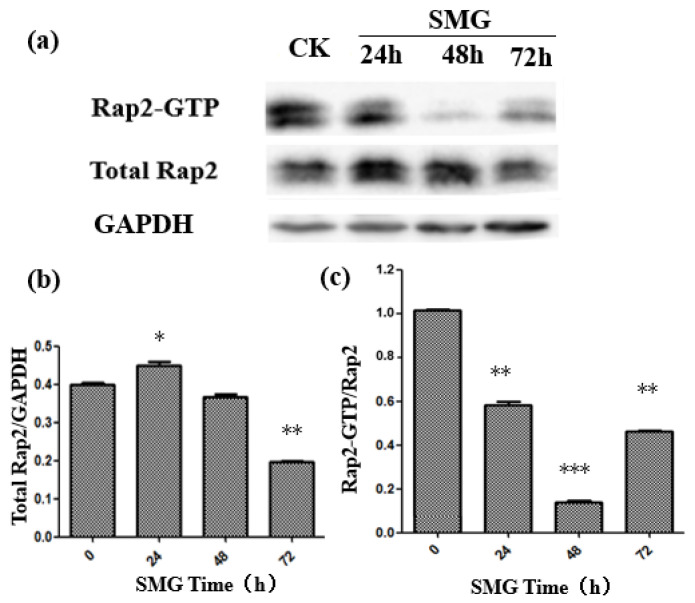
The activation of Rap2 was down-regulated by SMG. (**a**) Expression and activation of Rap2 after different times of SMG treatment were detected by Western blot and GST pull-down assay, respectively. (**b**) The relative Rap2 expression under SMG for 24, 48, and 72 h was calculated based on panel (**a**). (**c**) The relative activation of Rap2 under SMG for 24, 48, and 72 h was calculated based on panel (**a**). The statistical results shown represent the means (*n* = 3) vs. the control group (*n* = 3); * *p* < 0.05, ** *p* < 0.01, *** *p* < 0.001.

**Figure 4:**
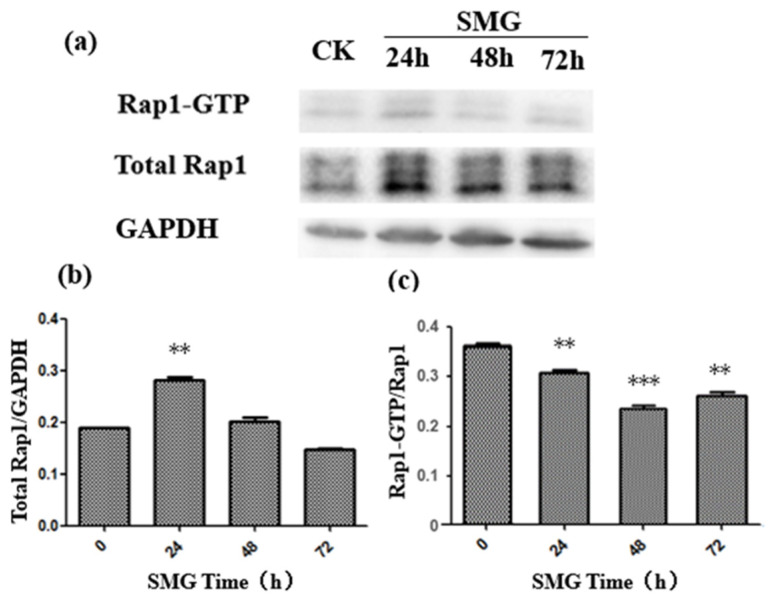
The activation of Rap1 was down-regulated by SMG. Expression and activation of Rap1 protein under SMG for 24, 48, and 72 h were detected by Western blot and GST pull-down assay, respectively. (**b**) The relative Rap1 expression under SMG for 24, 48, and 72 h was calculated based on panel (**a**). (**c**) The relative activation of Rap2 under SMG for 24, 48, and 72 h was determined based on panel (**a**). The statistical results shown represent the means (*n* = 3) vs. the control group (*n* = 3); ** *p* < 0.01, *** *p* < 0.001.

Rap small G proteins regulate the formation of endothelial cell adherens junctions [17]. To elucidate the mechanism of enhanced permeability of the HUVEC monolayer under SMG, the activity of both Rap1 and Rap2 was detected after SMG using a GST pull-down experiment. The activation of Rap1 was slightly decreased, while Rap2 activation was largely down-regulated with the lowest activation at 48 h treatment (Figure 3 and Figure 4). The activation levels of Rap1 and Rap2 were slightly recovered at 72 h treatment. However, the activation level of Rap2 in HUVEC cells under SMG was still greatly lower than the level of control. On the other hand, the level of expression of Rap1 and Rap2 showed a similar pattern with a slight increase at 24 h and a gradual recovery since 48 h SMG treatment.

### 2.3. Rap1GAP Was Down-Regulated under Simulated Microgravity

**Figure 5:**
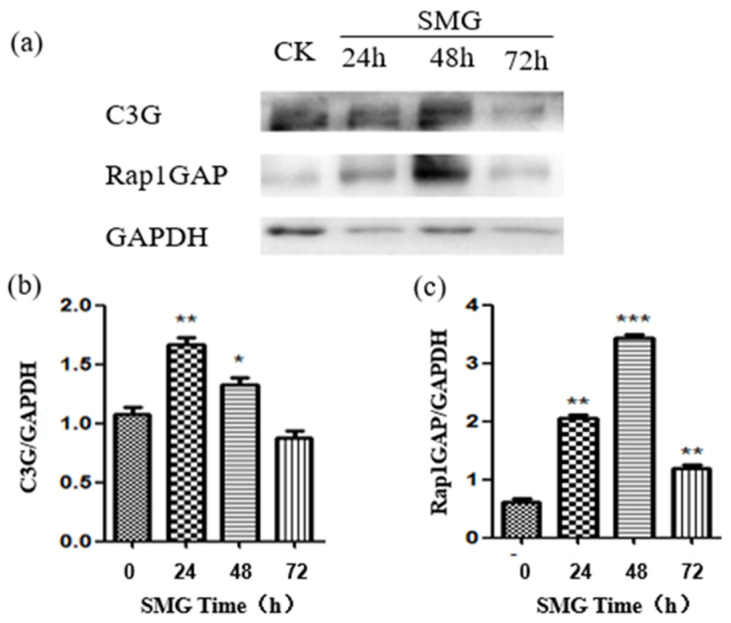
The expression of C3G and Rap1GAP protein under SMG was up-regulated. (**a**) The proteins of HUVEC cells were extracted after SMG treatment, and the expression of C3G and Rap1GAP were detected with Western blots using anti-C3G or anti-Rap1GAP specific antibody, respectively. (**b**,**c**) The relative expression of C3G and Rap1GAP under SMG for 24, 48, and 72 h was calculated based on intense assay of each band of protein. The statistical results shown represent the means (*n* = 3) vs. the control group (*n* = 3); * *p* < 0.05, ** *p* < 0.01, *** *p* < 0.001.

RapGEF1 or C3G, which is short for rap guanine nucleotide exchange factor 1, could up-regulate the activation of Rap1 and Rap2. Rap1GAPs down-regulate the activation of Rap1 and Rap2 [21]. To elucidate the mechanism of down-regulation of Rap2, both the activation of RapGEF1 and Rap1GAP were determined under microgravity. The results indicate that both C3G and Rap1GAP were up-regulated, but the pattern and intensity of their expression differed greatly (Figure 5). The expression of C3G reached its peak at 24 h, and C3G was up-regulated by 60% at that time under SMG. Meanwhile, the expression of Rap1GAP peaked at 48 h, and C3G was up-regulated by five times at that time under SMG. Comparing the expression pattern and intensity of C3G and Rap1GAP, the enhanced level of Rap1GAP should be dominant over C3G to regulate the activation of Rap1 and Rap2 at 24 h and 48 h treatment of SMG.

### 2.4. The Expression of VE-Cadherin and β-Catenin Was Down-Regulated under Simulated Microgravity

**Figure 6:**
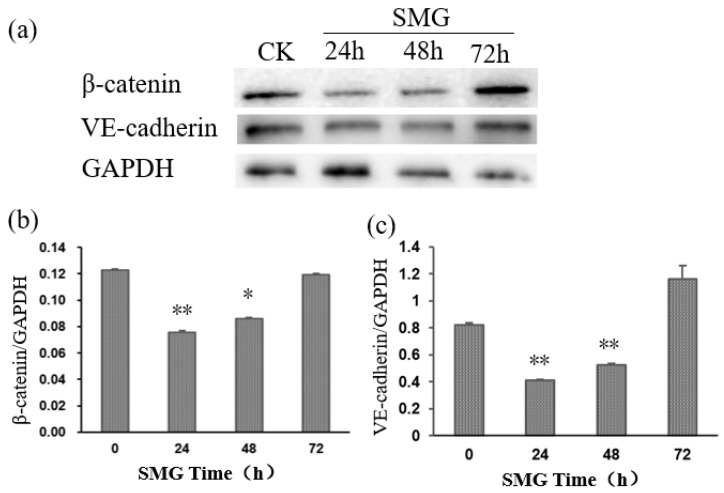
The expression of VE-cadherin and β-catenin was down-regulated under SMG. (**a**)The total proteins were isolated from HUVEC cells treated with SMG, and expression of β-catenin and VE-cadherin protein was detected by Western blot with anti-β-catenin or anti-VE-cadherin antibody, respectively. (**b**,**c**) The relative expression of β-catenin and VE-cadherin under SMG for 24, 48, and 72 h was calculated based on intense assay of each band of protein. The statistical results shown represent the means (*n* = 3) vs. the control group (*n* = 3); * *p* < 0.05, ** *p* < 0.01.

**Figure 7:**
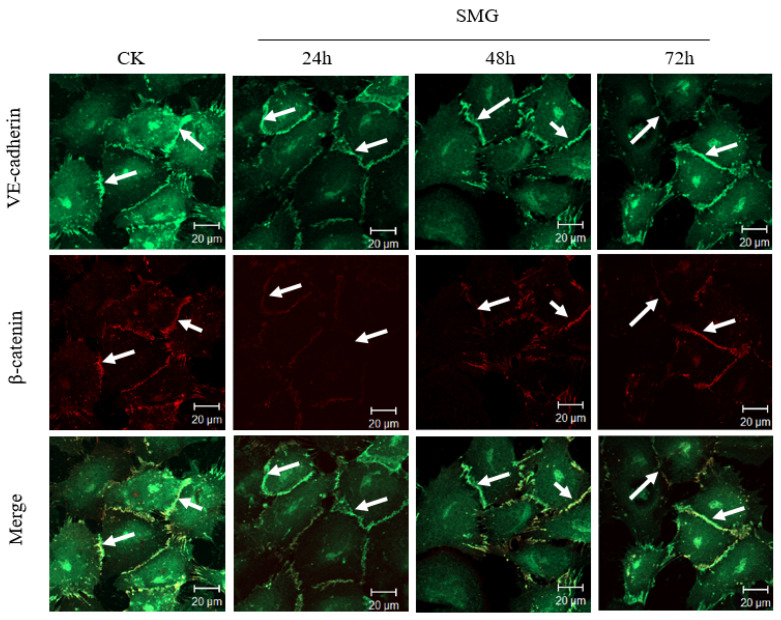
The expression and distribution of VE-cadherin and β-catenin in HUVEC cells under SMG. The upper panel: VE-cadherin in green; the middle panel: β-catenin in red; the lower panel: merged. The white arrows shows the location of VE-cadherin (first line), β-catenin (second line) and the co-localization of VE-cadherin and β-catenin. Scale bar = 20 µm.

As the increased permeability of the HUVEC monolayer may attribute to the altered expression or subcellular localization of VE-cadherin, the expression and distribution of VE-cadherin were detected by Western blotting and immunofluorescence assay after SMG (Figure 6 and Figure 7). As expected, the expression of VE-cadherin was significantly down-regulated with the lowest expression of VE-cadherin at 24 h after SMG. The expression of VE-cadherin was gradually recovered from 48 h to 72 h under SMG (Figure 6). The down-regulated expression of VE-cadherin was also confirmed by immunofluorescence using anti-VE-cadherin specific antibody (Figure 7). However, we could not find the change in the subcellular distribution pattern of VE-cadherin. Besides the membrane localization at cell–cell contact, VE-cadherin could be detected at the perinuclear region and cytosol of HUVEC cells both in control and SMG treatments. As β-catenin connects VE-cadherin to actin filaments through interaction with the intracellular domain of VE-cadherin and regulates the adherens junction formation between endothelial cells, we tested whether the expression and distribution of β-catenin were affected by SMG treatment using Western blotting and immunofluorescence assay with anti-β-catenin specific antibody (Figure 6 and Figure 7). The results indicate that, similar to VE-cadherin, the expression of β-catenin showed a pattern of an upward curve with the valley of VE-cadherin at 24 h SMG treatment. However, the extent of down-regulation of β-catenin by SMG was much higher than VE-cadherin. The expression of β-catenin was gradually recovered from 48 h and peaked at 72 h after SMG (Figure 6). The down-regulated expression of β-catenin was also confirmed by immunofluorescence using anti-β-catenin specific antibody (Figure 7). Although β-catenin mainly showed a membrane-localized pattern, the level of fluorescence intensity was much lower at 24 h SMG than that in the control cells.

### 2.5. The Distribution of Actin Filaments Was Modulated by Simulated Microgravity

**Figure 8:**
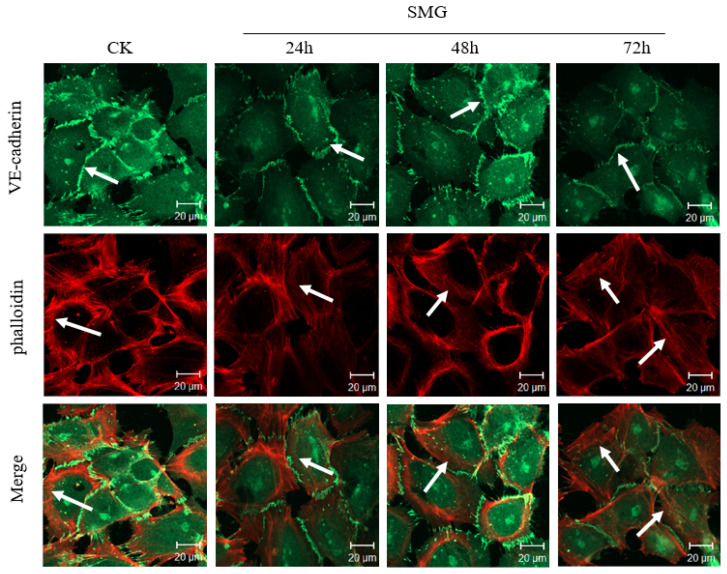
The expression and distribution of VE-cadherin and actin filaments in HUVEC cells under SMG. The upper panel: VE-cadherin stained in green with antibody against VE-cadherin; middle panel: actin filaments stained in red with phalloidin conjugated with Texas Red; the lower panel: merged. The white arrows in the first line show the localization of VE-cadherin. The white arrows in the second line show the localization of F-acting. The white arrows in the third line show F-acting localized beneath adherens junctions formed by VE-cadherin. Scale bar = 20 µm.

A cortical distribution of actin filaments under the cell–cell adherens junction forms through binding to the intracellular domain of VE-cadherin mediated by α-catenin, β-catenin, and p120-catenin and enhances the cell–cell interaction which contributes to decreased permeability through the endothelial cell layer. To detect whether SMG could affect the cortical distribution of actin filaments, immunofluorescence experiments were performed after SMG treatment of HUVEC cells using phalloidin staining (Figure 8). In contrast to the major cortical distribution of actin filaments under the cell–cell junctions in control HUVEC cells, the cortical distribution of actin filaments under cell–cell junctions was decreased. Instead, the stress fiber formation at the region of the cell bottom was apparent at 24 h and could still be seen until 72 h SMG treatment. These results suggest that the shift of cortical distribution to the stress fiber of actin filaments may be involved in the increased permeability through the HUVEC monolayer under SMG.

### 2.6. The Knock-Down of Rap1GAP Decreased the Permeability of HUVEC Monolayer under Simulated Microgravity

**Figure 9:**
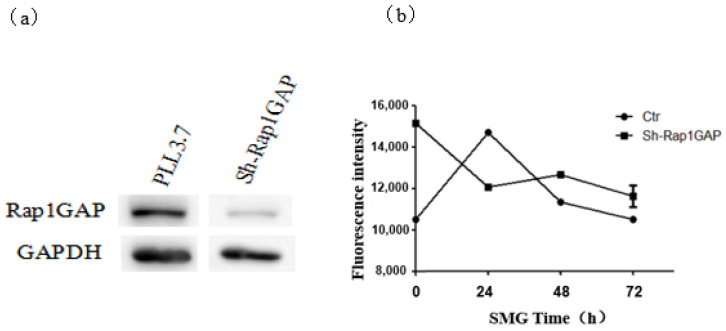
Rap1GAP interfering with cell line construction and detection. (**a**) Intracellular Rap1GAP interference efficiency. (**b**) The effect of simulated microgravity on the permeability of monolayer HUVEC cells after interfered with by Rap1GAP.

It has been reported that Rap2 signaling is activated during cell–cell junction formation and may be involved in the regulation of permeability of the endothelial cell monolayer. Rap1 GAP negatively regulates the activation of Rap2 by promoting the shift of the GTP-binding state to the GDP-binding state of Rap2 [20]. To address the role of Rap1GAP in the permeability of the HUVEC monolayer, Rap1GAPs were knocked down through shRNA expression. The permeability assay of monolayer HUVEC cells expressing shRNA specific to Rap1GAP was performed using a clinostat-based model under SMG (Figure 9). In contrast to the up-regulation of permeability of the HUVEC monolayer under the control condition, the knock-down of Rap1GAP decreased the permeability of the HUVEC monolayer under SMG, and the decreased permeability lasted until 72 h after SMG treatment. The results indicate that Rap1GAP could regulate the permeability of the HUVEC monolayer under SMG.

## 3. Discussion

It has been shown that microgravity has a variety of biological effects including an abnormal cytoskeleton and down-regulated mechanic sensing proteins on endothelial cells [38,39,40,41,42]. However, whether microgravity has effects on the physiological functions of the endothelium has not been addressed up until now.

HUVECs are human umbilical vein endothelial cells isolated from the endothelium of umbilical veins. They are well used to investigate the phenotype of the cell–cell junctions and trans-endothelium permeability for macromolecules and blood cells [33]. To address the physiological effects of SMG on the integrity of the endothelium, we established a HUVEC monolayer model based on 2D-clinostat rotation to monitor the change of permeability of the HUVEC monolayer under SMG. We found that the SMG largely increased the permeability of the HUVEC monolayer in a time-dependent manner. In addition, with the extension of SMG treatment, the permeability of the HUVEC monolayer recovered to the normal level at 48 and 72 h. Robert Szulcek et al. challenged an assay for the barrier integrity of the HUVEC monolayer under RPM-based microgravity or large-diameter centrifuge (LDC)-based hyper-gravity and showed that simulated microgravity leads to a decreased barrier integrity of the HUVEC monolayer, although the mechanism for the change in endothelial barrier integrity has not been investigated [39].

It has been reported that Rap1 and Rap2 antagonistically regulate the integrity of adherens junction between endothelial cells. Rap1 promoted the barrier resistance of the HUVEC monolayer, which was inhibited by Rap2 by an electrical cell impedance sensing (ECIS) assay [39]. In response to microgravity stress, in HUVEC cells, the expression of a large number of genes is drastically changed, which are mainly involved in cell adhesion, cell apoptosis, and cell cycle processes [41]. In addition, SMG affects the complexity of intracellular skeletal proteins and induces cytoskeletal rearrangement [42]. These findings suggest that Rap1 and Rap2 may respond to the simulated microgravity and thus regulate the adherens junctions between monolayer HUVEC cells under microgravity.

In this study, we found that the expression level of Rap1 and Rap2 proteins in HUVEC cells increased after 24 h of SMG treatment but decreased gradually to the basal level or to an even lower level than the control with regard to Rap2. On the contrary, the activation of Rap1 and Rap2 under SMG treatment decreased significantly, reaching the lowest value at 48 h, and then recovered partially. The decline rate of Rap2 reached 80%, which was much higher than the 35% decrease of Rap1 activation, suggesting that Rap2 plays a major biological role in response to SMG stress. Although there is still no direct evidence to support that Rap2 signaling could positively regulate the formation of adherens junctions up until now, the trans-dimerization of JAM-A, a tight-junction-associated protein, up-regulates the activation of Rap2 and further participants in the positive regulation of cell adherens junction in HEK293 cells [43,44].

As a negative regulator for the activation of both Rap1 and Rap2, Rap1GAP down-regulation in colon cancer cells inhibited the formation of adherens junction between cells but enhanced the adhesion of cells and extracellular matrix [45]. Therefore, the biological role of Rap1GAP should be dependent on the cell context and intercellular environment. Here, we found that the expression of Rap1GAP increased significantly in response to SMG treatment, peaking at 48 h after SMG. Meanwhile, the expression of C3G (also named Rap1GEF1) also showed a significant increase under SMG, with the highest level at 24 h SMG treatment. Meanwhile, it cannot be ignored that the expression level of Rap1GAP at 48 h SMG treatment increased five times compared to the control, which was much higher than the 50% increase of C3G at 24 h SMG treatment, suggesting that Rap1GAP plays a dominant role in regulating the activation of Rap2 under SMG treatment.

The formation of adherens junction is facilitated by the trans-interaction of VE-cadherin between endothelial cells and formation of the cortical actin filament bundle beneath the plasma membrane through connecting to the intracellular domain of VE-cadherin through interaction with β-catenin which will strengthen the adherens junction [7,18]. In these results, the increased permeability of a monolayer of HUVECs under SMG should be related to the loosening of adherens between HUVEC cells. This result shows that the expression of VE-cadherin and β-catenin in HUVEC cells decreased significantly under SMG treatment, while their intracellular localization did not change significantly (Figure 6 and Figure 7). Therefore, the increased permeability of the HUVEC monolayer resulted from SMG might just relate to the down-regulation of VE-cadherin and β-catenin in quantity but not the regulation of its intracellular distribution. However, our results do not rule out the mechanism of change at the posttranslational modification of VE-cadherin and β-catenin under SMG.

The increase in Rap1GAP expression under SMG is accompanied by significant down-regulation of Rap1 and Rap2 activity, indicating that the Rap1GAP-Rap axis may play a key role in the integrity of the HUVEC monolayer. Although there were reports showing that both Rap1 and Rap2 could regulate adherens junction between endothelial cells, the reports regarding the role of Rap2 in adherens junction are still lacking and rather controversial [34,41]. In normal conditions, the permeability of monolayer HUVEC cells expressing Rap1GAPshRNA was higher than that of cells expressing scrambled shRNA (Figure 9). The importance of Rap1GAP in cell–cell adhesion is also reported in research on carcinoma cells: deletion of Rap1GAP prevented the formation of adherens junctions in colorectal carcinoma indicating that Rap1GAP is necessary for the formation of adherens junctions at a basal level [32,46]. However, compared to the cells expressing scrambled shRNA, the increase of permeability in the HUVEC monolayer induced by SMG was significantly inhibited by the interference of Rap1GAP, and this inhibition lasted until 72 h after SMG. To elucidate whether decreased activation of Rap1 or Rap2 could contribute to the increased permeability of the HUVEC monolayer under SMG, acting downstream of Rap1GAP, the activation of Rap1 and Rap2 in HUVEC cells expressing Rap1GAPshRNA or the control shRNA was detected after SMG (Figure S1). The activation of both Rap1 and Rap2 was similar to the grand level, indicating that the up-regulation of the permeability of the HUVEC monolayer by Rap1GAP under SMG was mediated by the down-regulation of Rap1 and Rap2.

In conclusion, by establishing a model to monitor the permeability of the HUVEC monolayer under SMG, we found that SMG significantly increased the permeability of the HUVEC monolayer. The mechanism underlying the up-regulated permeability may involve the small G protein Rap signaling pathway as the significant up-regulation of Rap1GAP and down-regulation of Rap2 and Rap1 were confirmed under SMG. Our findings provide new cues that connect the mechanic sensing signal to the increased permeability of the HUVEC monolayer through down-regulating cell–cell adherens junctions.

## 4. Materials and Methods

### 4.1. Cultures of HUVEC

HUVEC cell line was used for the present study and was purchased from ATCC. HUVEC cells were cultured in DMEM (Corning, Shanghai, China) with 20% (*v*/*v*) fetal calf serum (FBS, Gibco-BRL, Shanghai, China), supplemented with ECGS (MACGENE, Shanghai, China).

### 4.2. Cell Culture in Rotating Culture Vessel

Rotating wall vessel (2D-RWVS) was originally developed by China Astronaut Research and Training Center. HUVEC cells were seeded over a cover slip (226 × 266 × 0.5 mm) and cultured on the slip for 24 h. The slip was then transferred into culture vessel of 0.04 m in diameter filled with growth medium. According to equipment manual and reference, the samples were rotated around the horizontal axis at 30 rpm to simulate microgravity effect (SMG) [47,48]. The cells cultured in the RWVS vessel without rotation were considered as the untreated control (static control) group.

To avoid the possibility that changes in protein expression over time might affect the results of the experiment, our experiment was designed as follows:

After the cells were seeded over a cover slip, the simulated microgravity experiments were conducted after 24th hour, 48th hour, and 72nd hour, respectively, and those who had not been simulated-microgravity-treated were control group. Samples of each group were collected at 96 h, and materials with microgravity for 72, 48, 24, and 0 h (ground control) were obtained. Because the culture time of each group was 96 h, the difference in protein expression was avoided.

### 4.3. Construction of Interference Vector

The oligos used in experiment are listed in Table 1 as follows.

h-*Rap1GAP*-s and Sh-*Rap1GAP*-a were mixed in annealing buffer and annealed by heating to 95 °C for 5 min, followed by a slow cooling to RT. pLentilox3.7Rap1GAPshRNA vector was constructed by inserting the annealed oligos to the HpaI-digested site of pLentilox3.7.

### 4.4. Virus Packaging and Infection

293T cells were grown to sub-confluence with DMEM medium supplemented with 10% FBS and cotransfected by pLentilox3.7shRap1GAP or Lentilox3.7-scramble-shRNA, pCMV-VSV-G), and pCAG-dR8.9. Forty-eight hours after transfection, the culture supernate was collected and filtered with filter of 0.45 μm to prepare the virus suspension. Then, HUVEC cells were grown to 60% confluency in 12-well plates and infected with the premixed virus suspension, M199 medium, and polybrene at a rate of 500:250:0.5. The culture suspension was replaced with fresh M199 medium supplemented with 20% FBS and ECGS 24 h after infection.

### 4.5. Screening of HUVEC Cells Expressing shRNAs

HUVEC cells expressing Rap1GAPshRNA or scrambled shRNA were collected and diluted 48 h after infections to a 96-well plate to ensure that the number of cells in each well was 1–2 cells. When the single clone grew to 50% confluence, the cells were replated to 24-well plate and grown to confluence. Then the expression of Rap1GAP was detected by Western blot to determine the interference efficiency.

### 4.6. HUVEC Monolayer Permeability Model

A total of 4 × 10^4^ HUVEC cells were seeded on a transwell chamber with pore of 3 μm in diameter and allowed to grow until a uniform monolayer was formed by HUVEC cells. The chamber was then transferred to a culture vessel (Figure 1) containing M199 medium supplemented with 20% FBS and 0.5% ECGS. The culture vessels set with HUVEC monolayer grown on the surface of filter of the chamber were set on 2D-clinostat and let to rotate horizontally to simulate the microgravity condition. The vessels set with HUVEC monolayer grown on the surface of filter of the chamber were set on 2D-clinostat and allowed to rotate vertically. The chambers were removed from the vessels 24, 48, and 72 h after rotation of vessels in 2D-clinostat machine to a 24-well plate. The medium in the upper room of the chamber was discarded, and the chamber was washed with Hanks’ Buffer and added with 100 μL of 2.5 mg/mL of FITC-dextran (MW4000, Sigma, Shanghai, China). The lower room of the chambers set on 24-well plate was added with 500 μL Hanks’ Buffer. The chambers were incubated at 37 °C with 5% CO_2_ for 30 min in a dark condition. The chambers were removed from 24-well plate, 50 μL of solution in the lower room of the chamber was removed to 96-well plate, and the intensity of fluorescence for FITC in the lower room was measured in a microplate reader (Bio-Rad). The permeability assay of HUVEC monolayer was repeated three times for each time point. Meanwhile, the control experiment was performed by using the same protocol with different concentration of FITC-dextran (MW4000). The standard curve was plotted after monitoring intensity of fluorescence of the lower chamber at each concentration of FITC-dextran (Figure 1).

### 4.7. Immunofluorescence Analysis

HUVEC cells were fixed at room temperature with 4% PFA (Sigma, St. Louis, MO, USA) for 15 min, permeabilized in 1% (*v*/*v*) TritonX-100 (AMERSCO, Washington, DC, USA) for 10 min, and blocked with 5% BSA (Sigma, St. Louis, MO, USA) for 60 min. The cells were incubated overnight at 4 °C with mouse anti-VE-cadherin, anti-β-catenin, or Alexa568-conjugated phalloidin (1:1000, Sigma, St. Louis, MO, USA), diluted with PBS, and were incubated at 37 °C for 1.5 h with FITC-conjugated goat anti-mouse IgG (1:100, Zhongshan Biotechnology Co. Ltd., Beijing, China) diluted with PBS. The cells after washing were observed with confocal microscope (Zeiss, Munich, Germany).

### 4.8. Western Blot

HUVEC cells treated with SMG or the control condition were lysed with RIPA buffer (150 mM sodium chloride, 1% Triton X-100, 0.5% sodium deoxycholate, 0.1% sodium dodecyl sulfate, 50 mM Tris, pH 8.0). Aliquots of the protein were separated with 12% SDS-PAGE and transferred onto PVDF filters. The membranes were blocked with PBS-T containing 5% milk and then incubated overnight at 4 °C with primary antibody (Rap1 (Abcam, Cambridge, UK), Rap2 (Santa Cruz, CA, USA), C3G (Santa Cruz, CA, USA), Rap1GAP (Abcam, Cambridge, England), VE-cadherin (Abcam, Cambridge, UK), β-catenin (Santa Cruz, CA, USA) or GAPDH (Sigma, Shanghai, China), and the PVDF filters were incubated for 1 h with HRP-conjugated secondary antibody. ECL reaction (Sangon Biotech, Shanghai, China) was used to detect the signals. The relative expressions of detected protein were normalized to amount of GAPDH.

### 4.9. GST Pull-Down Assay for Activation of Rap1 and Rap2

HUVEC cells under SMG treatment or control condition were lysed in Ral buffer (1% NP-40, 10% Glycerol, 50 mM Tris-HCl (pH7.4), 150 mM NaCl, 2.5 mM MgCl_2_, 10 mM NaF, 1 mM Na_3_VO_4_, 1 μg/mL Leupeptin, 1 μg/mL Aprotinin, 1 mM PMSF). The cell lysates were centrifuged at 12,000 rpm for 15 min at 4 °C. The activation of Rap1 and Rap2 in the supernatant fraction was carried out with GST pull-down assay as described previously [46].

### 4.10. Data Analysis

All the experiments were repeated at least three times. Statistical significance was performed using Student’s *t*-test. Differences were considered to be significant at *p* < 0.05.

## 5. Conclusions

According to the experimental results obtained, the following conclusions are drawn:

(1) The simulated microgravity effect can affect the expression level and activation state of Rap1 and Rap2 in the cell, and the SMG condition will affect the adhesion and connection of monolayer HUVEC cells. With the extension of the treatment, the strength of the adhesion and connection between the cells would decrease and then recover, reaching the weakest state at 24 h of SMG.
(2) The simulated microgravity effect has an impact on the expression of four proteins that have an impact on cell adhesion and junction in HUVEC cells: Rap1GAP, C3G, VE-cadherin, and β-catenin. Under the SMG effect, the expression of Rap1GAP and C3G proteins would increase and then decrease, while that of VE-cadherin and β-catenin proteins would decrease and then recover.
(3) Under the simulated microgravity effect, the two proteins Rap1GAP and VE-cadherin would affect the adhesion and connection state of the monolayer HUVEC cells. When the intracellular Rap1GAP was interfered with, the intercellular adhesion would slightly enhance under the SMG effect. When intracellular VE-cadherin was interfered with, the adhesion and junction between cells under the effect of SMG would slightly enhance and then recover.

## Figures and Tables

**Table 1:** Oligo sequences.

Gene Name	Primer Sequence
Sh-*Rap1GAP*-s	5′-TGCAATGTGGTATTTCAAGAGAATAGAATAGAACCGATCCACATTGCTTTTTTC-3′
Sh-*Rap1GAP*-a	5′-TCGAGAAAAAAGCAATGTGGATCGGTTCTATTCTCTTGAAATAGAACCGATCCACATTGCA-3′

## Data Availability

All data are available in this publication and in the Supplementary Materials.

## Supplementary Materials

The following are available online at https://www.mdpi.com/article/10.3390/ijms23020630/s1.

## Abbreviations

SMG	simulated microgravity effect
CK	check or control
GEF	guanine nucleotide exchange factor
kDA	kilodalton
DAPI	4′,6-diamidine-2-phenylindol
RWVS	rotating wall vessel
PFA	paraformaldehyde
PBS	phosphate buffer saline
RIPA	radio immunoprecipitation assay
HRP	horseradish peroxidase
GAPDH	glyceraldehyde-3-phosphate dehydrogenase
