# Supplementary material for: Simulated Microgravity Increases the Permeability of HUVEC Monolayer through Up-Regulation of Rap1GAP and Decreased Rap2 Activation
Source: Int J Mol Sci. 2022 Jan 6;23(2):630. doi: 10.3390/ijms23020630 (PMC8776081; doi:10.3390/ijms23020630)
Supplement: Supplementary file 1 [file ijms-23-00630-s001.zip › ijms-1385163-supplementary.pdf]

(a) (b)

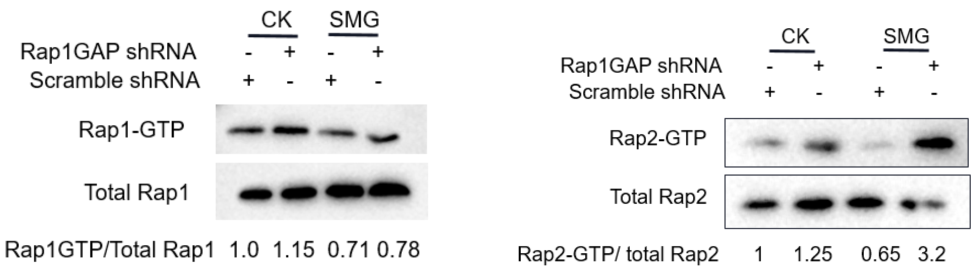

Figure S1 The activation of Rap1(a) and Rap2 (b)in HUVEC cells expressing Rap1GAPshRNA or the control shRNA.
